# Health care seeking behaviour and financial protection of patients with hypertension: A cross-sectional study in rural West Bengal, India

**DOI:** 10.1371/journal.pone.0264314

**Published:** 2022-02-25

**Authors:** Sandipta Chakraborty, Rajesh Kumar Rai, Asit Kumar Biswas, Anamitra Barik, Preeti Gurung, Devarsetty Praveen

**Affiliations:** 1 Institute of Public Health, Kalyani, West Bengal, India; 2 Department of Preventive and Social Medicine, All India Institute of Hygiene and Public Health, West Bengal, India; 3 Society for Health and Demographic Surveillance, Suri, West Bengal, India; 4 Department of Global Health and Population, Harvard T.H. Chan School of Public Health, Boston, Massachusetts, United States of America; 5 Department of Economics, University of Goettingen, Goettingen, Germany; 6 Centre for Modern Indian Studies, University of Goettingen, Goettingen, Germany; 7 Suri District Hospital, Suri, West Bengal, India; 8 Medecins Sans Frontieres, Manipur, India; 9 Primary Health Care Research, The George Institute for Global Health, Hyderabad, Telangana, India; 10 Faculty of Medicine, University of New South Wales, Kensington, NSW, Australia; 11 Prasanna School of Public Health, Manipal Academy of Higher Education, Manipal, India; Murdoch University, AUSTRALIA

## Abstract

**Background:**

Elevated blood pressure or hypertension is responsible for around 10 million annual deaths globally, and people residing in low and middle-income countries are disproportionately affected by it. India is no exception, where low rate of treatment seeking for hypertension coupled with widespread out-of-pocket payments (OOPs) have been a challenge. This study assessed the pattern of health care seeking behaviour and financial protection along with the associated factors among hypertensive individuals in rural West Bengal, India.

**Method and findings:**

A cross-sectional study was conducted in Birbhum district of the state of West Bengal, India, during 2017–2018, where 300 individuals were recruited randomly from a list of hypertensives in a population cohort. Healthcare seeking for hypertension and related financial protection in terms of–OOPs and expenses relative to monthly per-capita family expenditure, were analysed. Findings indicated that 47% of hypertensives were not on treatment. Among those under treatment, 80% preferred non-public facilities, and 91% of them had wide-spread OOPs. Cost of medication was a major share of expenses followed by transportation cost to access public health care facility. Multivariable logistic regression analysis indicated longer duration of disease (adjusted odds ratio (aOR): 5.68, 95% Confidence Interval (CI) 1.24–25.99) and health care seeking from non-public establishment (aOR: 34.33, CI: 4.82–244.68) were associated with more incident of OOPs. Linear regression with generalized linear model revealed presence of co-morbidities (adjusted coefficient (aCoeff)10.28, CI: 4.96,15.61) and poorer economic groups (aCoeff_poorest_ 11.27, CI 3.82,18.71; aCoeff_lower-middle_ 7.83, CI 0.65,15.00 and aCoeff_upper-middle_ 7.25, CI: 0.80,13.70) had higher relative expenditure.

**Conclusion:**

This study suggests that individuals with hypertension had poor health care seeking behaviour, preferred non-public health facilities and had suboptimal financial protection. Economically poorer individuals had higher burden of health expenditure for treatment of hypertension, which indicated gaps in equitable health care delivery for the control of hypertension.

## Introduction

Globally, non-communicable diseases (NCDs) contribute to a major share of the disease burden, where countries with differential level of development and varied phases of epidemiological transition have witnessed a significant rise in overall morbidity and mortality from NCDs [1–3]. Among all NCDs, cardiovascular diseases (ischaemic heart disease and stroke) are listed as the major cause of death worldwide, with hypertension (commonly defined as a systolic blood pressure ≥ 140 or diastolic blood pressure ≥ 90) being the most important risk factor causing significant amount of premature deaths globally [4, 5]. According to World Health Organization estimations, the number of adults aged 30–79 years having hypertension is 1.28 billion worldwide. Majority of them (two-thirds) are from low-and middle-income countries (LMICs) and only less than half are taking treatment [5]. Despite the high burden of hypertension, health system responses like health service delivery, health information and health financing for hypertension is suboptimal, especially in LMICs [6–10]. Evidence suggests that people seeking health care for NCDs bear significant and unjustified financial burden characterised by huge out-of-pocket payments (OOPs), often leading to irregular and absence of treatment seeking due to financial difficulties [10, 11]. In addition, studies show that overall health care seeking behaviour for blood pressure management is low and shared among public and non-public facilities [12, 13].

In India, between one-quarter to one-third of adults, aged 18 years or more, have hypertension. This is a major public health concern and threat to Indian healthcare system [14–16]. In the year 2010, to combat the emerging NCDs and its risk factors, the federal Indian government introduced the National Programme for Prevention and Control of Cancer, Diabetes, Cardiovascular Diseases and Stroke (NPCDCS) with hypertension and diabetes as the main focus areas. In addition, in 2017, the government launched the National Health Policy targeting 25% reduction in premature mortality occurring from cardiovascular diseases, cancer, diabetes or chronic respiratory diseases by 2025 [17, 18]. But the impacts of these program and policy level initiatives are not evaluated extensively. The main focus for research on hypertension in India is primarily on the risk factors of hypertension while few actually explored the health care utilization and service expenses and overall health system performance among hypertensive individuals, as evidenced from the PubMed/MEDLINE database search [19–22]. From the perspective of health system strengthening and population health management, understanding the local preferences and health system capacity is essential. Considering dearth of literature in this field, a study, Capacity of Health Systems to combat the Emergence of Hypertension (COHESION), was designed to assess the status of blood pressure control, health care seeking and financial protection among hypertensive individuals along with the health system responsiveness towards them. Here, we present a component of COHESION study to elicit the pattern of health care seeking, determine financial protection and its associated factors among patients with hypertension in rural West Bengal. The study aimed to understand how background socio-demographic characteristics, co-morbid conditions, health seeking pattern could be associated with financial protection related to heath care seeking and whether the issue of fair financing distributed with equity among the participants.

## Materials and methods

### Study setting, design and sampling

COHESION study is a population-based cross-sectional study, conducted between November 2017 and February 2018 in a population cohort of Birbhum Population Project (BIRPOP), a health and demographic surveillance system (HDSS) functioning under the ambit of Society for Health and Demographic Surveillance (http://www.shds.co.in/), located in the Birbhum district of the state of West Bengal, India,. BIRPOP spreads over four administrative blocks (namely Suri I, Sainthia, Mohammad Bazar and Rajnagar) out of a total of 19 blocks in district Birbhum. At its inception in 2008, BIRPOP included a sample of over 12,000 households selected by multistage stratified sampling method and has been periodically collecting information on indicators related to public health and demography. Until inception of the present study, BIRPOP had completed three rounds of follow-up surveys, in 2008–09, 2012–13, and 2016–17 [23]. COHESION study was based on BIRPOP’s 2016–17 survey where blood pressure was measured for 12,255 individuals aged ≥ 18 years. Those recorded with high blood pressure (systolic blood pressure (SBP) ≥140 mm of Hg and/or diastolic blood pressure (DBP) ≥90 mm of Hg) or reported taking anti-hypertensive medication of any form were included in the hypertensive cohort [23, 24]. Details about the blood pressure measurement survey at BIRPOP has previously been published elsewhere [25]. From the list of all hypertensive individuals, 310 were selected by simple random sampling for this study. Sample size was calculated using CDC Epi-info^TM^ version 7.2, assuming 50% prevalence for hypertension control among all hypertensives, 7.5% of error and confidence interval of 99%. With the addition of 5% non-response rate, final sample size was 310 individuals of which 300 interviews were conducted. Terminally ill and mentally challenged individuals, diagnosed by a physician, were not considered for participation in the study. In case, if more than one individual had been selected from same family, it was planned to replace the latter one. Data were collected by trained surveyors with pre-designed and pretested questionnaire using Computer Assisted Personal Interview (CAPI) technique [26]. A rigorous protocol for survey monitoring was followed to assure the quality of the data being collected.

### Outcome measurement

**Fig 1:**
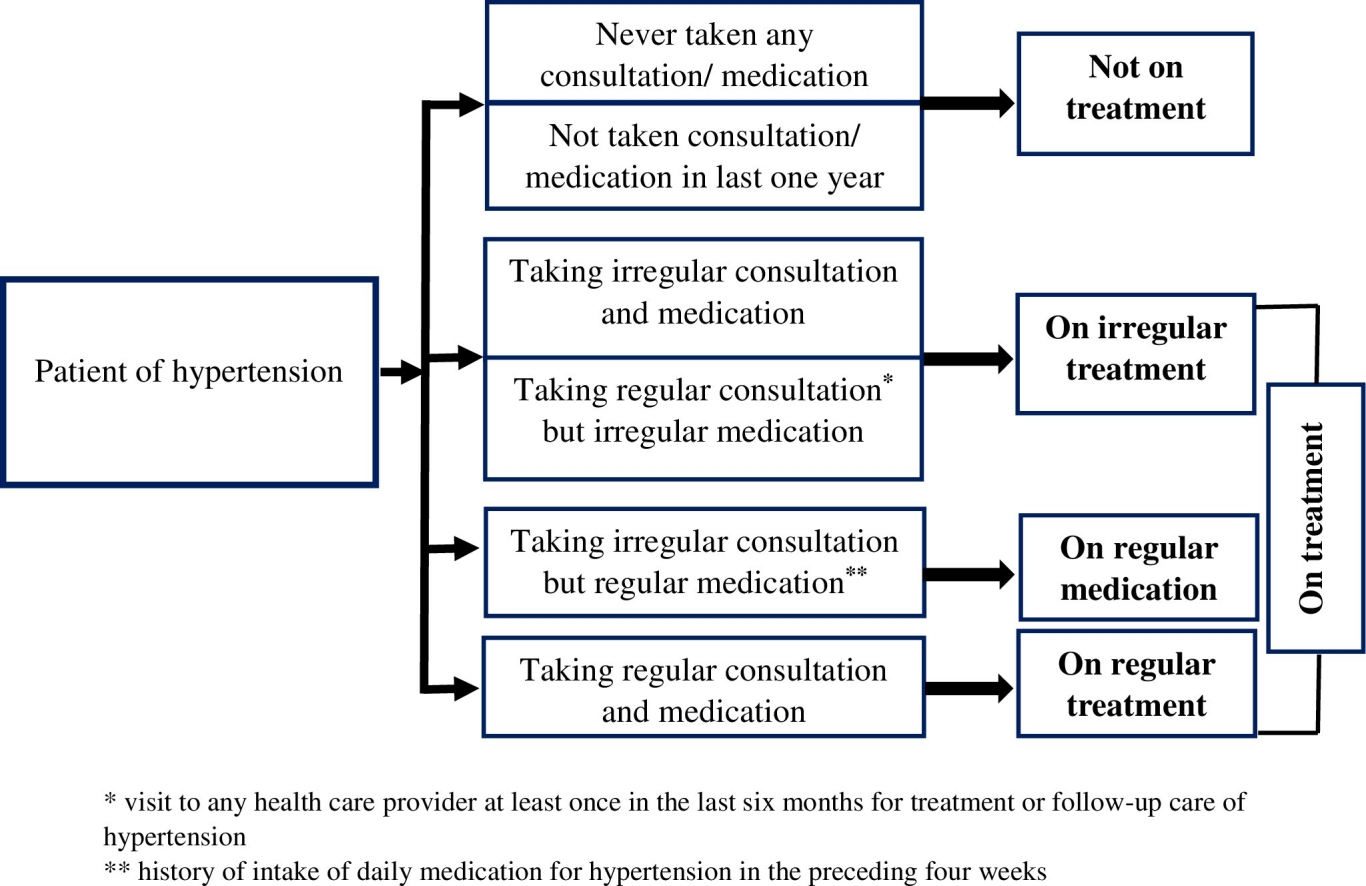
Construct of health care seeking behaviour for hypertension among the participants.

Health care seeking for chronic NCDs, like hypertension comprises of a complex dynamic interplay between medicine intake, visit to health care professional and regularity of both the components within a reference time period (**Fig 1**). To understand the health care seeking behaviour, patients were asked if they were taking any medication for blood pressure control and have been visiting any healthcare provider. Patients with a history of intake of daily medication for hypertension in the preceding four weeks were considered to be on regular medication. Those with a history of visit to any health care provider at least once in the last six months for treatment or follow-up care of hypertension, were considered to have regular medical consultation. Patients who had both of the above (regular medication and regular medical consultation) were labelled as having ‘regular treatment for hypertension’. Those who reported only regular medication but not regular medical consultation was identified as having ‘regular medication only’. Patients currently not on any medication or consultation for last one year or never sought any treatment for hypertension, were labelled as ‘not on treatment’. The rest were categorised as ‘patients on irregular treatment’ (**Fig 1**).

Two outcomes in relation to cost of treatment, were analysed in this study–i) Out-of-pocket payments (OOPs), and ii) expenses relative to monthly per-capita family expenditure, henceforth termed as ‘relative expenditure’. Considering the varied practice of health care seeking behaviour, expected monthly OOPs were calculated assuming an ideal month when complete health care (consultation by a physician and regular medicine intake) was sought. Thus, total expected OOPs for a month were calculated considering summative expenses that included expenses paid for medical consultation, transport and others, like food, lodging etc. during the consultation in the last medical visit, and cost of blood pressure lowering medication if taken for a month. Monthly per capita expenditure (MPCE) was calculated as monthly total consumer expenditure in a household over all items of consumption divided by the household size (total number of persons in the household) and was used as the proxy measure of the economic status [27]. Based on the MPCE, the participants were divided into four quartile classes and categorised into relative economic groups: poorest, lower-middle, upper-middle and richest class. Relative expenditure for an individual for a month was defined as percentage of MPCE incurred for OOPs [19].

### Covariates

Based on existing literature from developing countries, a range of potential covariates were considered.

#### Socio-demographic characteristics

This included age in completed years (later categorised based on tertile distribution- <50, 50–63, >63), gender (female and male), educational attainment (secondary and above, upper primary, primary, and illiterate/below primary), social group (other backward classes, scheduled castes/ scheduled tribes and others), religion (Hinduism and Islam), civil status (living with partner, and not living with partner), employment status (service/business, labourer, homemaker/retired/student, and unemployed), and economic status based on MPCE quartile distribution (high, upper-middle, lower-middle, and poor).

#### Hypertension related variables

This included duration of hypertension (<5 years, ≥5 years, and not sure/don’t know), co-morbidity (no and yes), regularity of treatment of hypertension (as elaborated before in Fig 1), type of health facility accessed (public, i.e. all government and semi-government health facilities and non-public), and healthcare provider like, public physician, private physician, AYUSH (*Ayurveda*, Yoga and Naturopathy, *Unani*, *Siddha* and Homoeopathy) doctor, and informal health care practitioner (Quack) [28]. Comorbidity refers to self-report about any of the diseases like diabetes, dyslipidaemia, chronic kidney disease or cardiovascular disease in addition to hypertension.

### Statistical analysis

**Fig 2:**
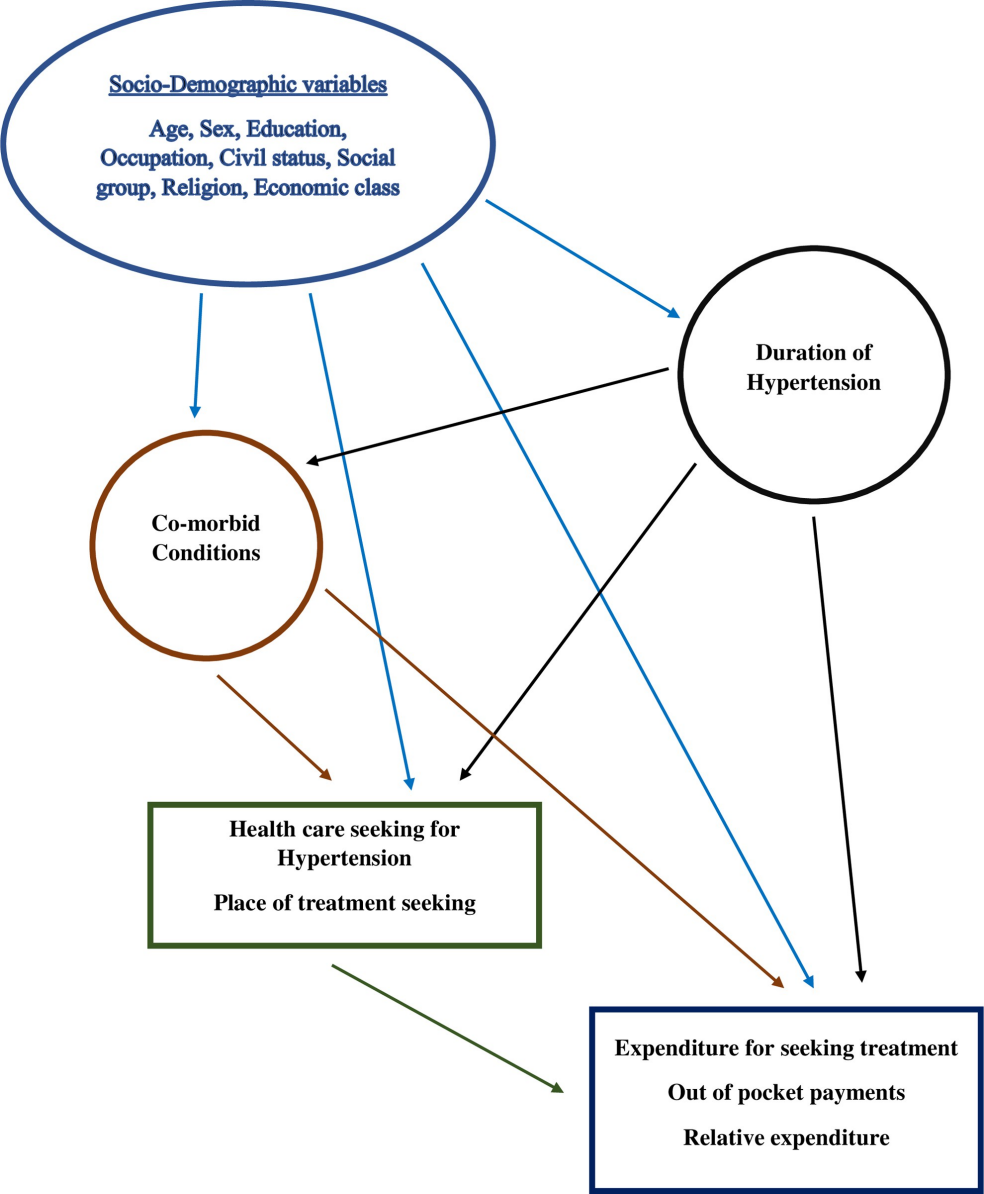
Directed acyclic graph: Illustrating associations between variables.

Bivariate and multivariable analyses were performed to attain the study objectives. Means and proportions were presented with 95% confidence intervals. Based on existing evidences, a Directed Acyclic Graph (DAG) was developed, using causal diagram theory [29]. The DAG illustrated the pathways of possible association with directed arrows, between the variables incorporated in the study. This diagram served as an overall theoretical framework along with a guide for constructing various regression models used in the analysis (**Fig 2**). Binary logistic regression was deployed to understand the predictors of OOPs, whereas linear regression by generalized linear models (GLM) was used to assess the relative expenditure. Measures of association were presented as odds ratio (OR) with 95% confidence interval (CI) with value “1” as the null point. GLM was preferred because of abundance of zero values in relative cost data and a possible non-parametric distribution of the relative expenditure [30]. With the linear modelling, the association is expressed with the estimated coefficient (Coeff) and associated 95% CI. “Zero” was considered as the null point. Data analysis were carried out using a statistical package—Stata, version 12.0. Alpha level was assumed as 0.05, *p* value ≤0.05 was considered to interpret the significance of observed association in general. Although qualitative interpretation based on *p* value (significant/non-significant based on conventional cut off) was judged cautiously, keeping with the sample size, study design, limitations and considering the effect size based on respective 95%CI of the measures of association.

### Ethics statement

Ethical approval was granted by institutional review board of Society for health and Demographic Surveillance. Written informed consent was obtained from all participants prior to enrolment in the study. Irrespective of their participation status, all, who were approached to participate in the study were provided with a leaflet on healthy lifestyle, health education related to hypertension and other NCDs written in local language.

## Results

**Fig 3:**
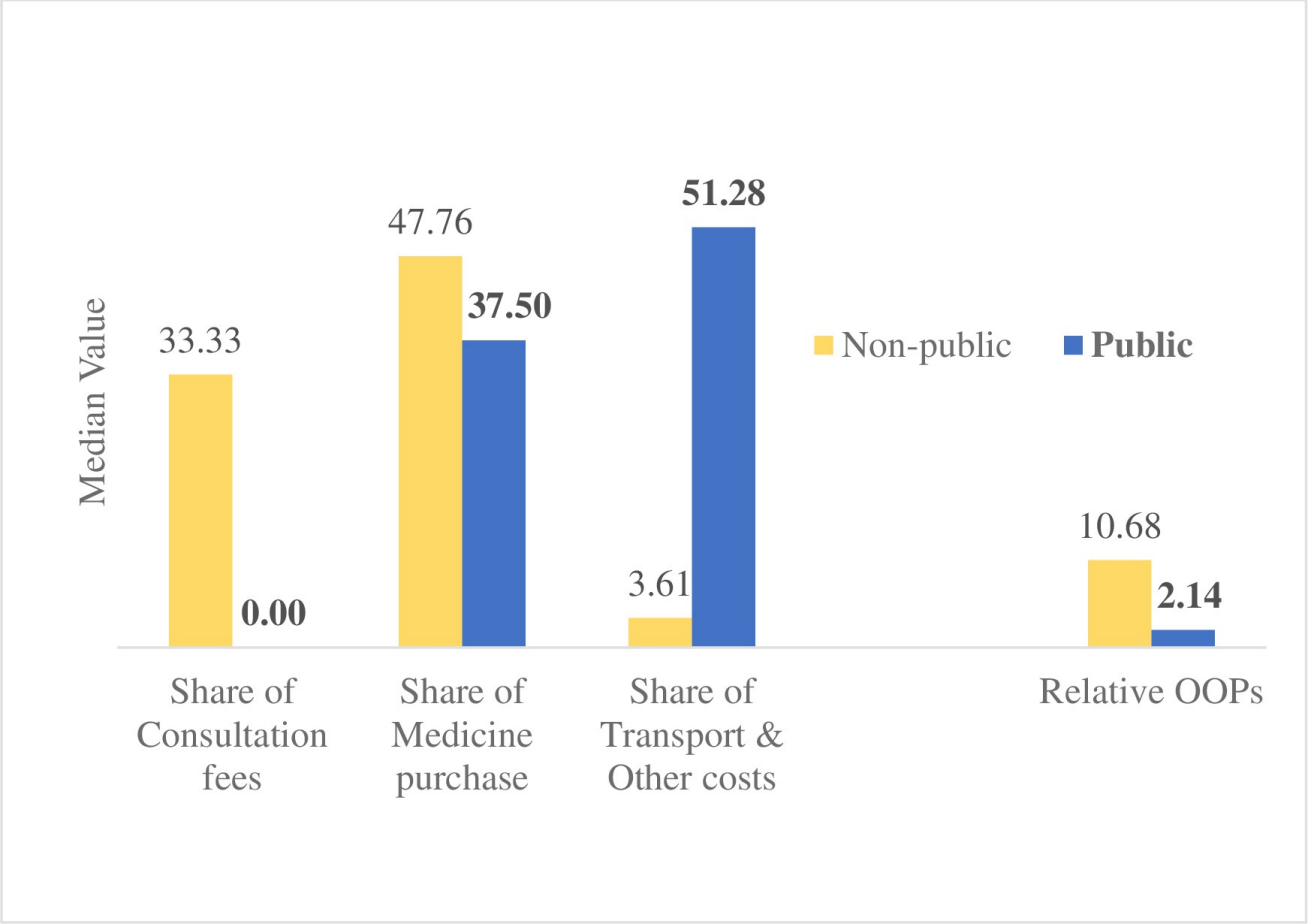
Median of OOPs share (%) and relative expenditure across the public and non-public health establishments (N = 159).

In total, 310 were approached to participate in this study, and 300 finally participated. **Table 1** outlines the descriptive characteristics of all the participants. The mean age of the participants was 55.99 ± 12.46 years. More than half of the participants were female and were illiterate or had not completed their primary education. Majority of the participants were Hindus and homemaker/retired/students by profession. Over 35% (n = 106) of participants had hypertension for ≥5 years, and 20% (n = 60) had a co-morbid condition. Over 47% (n = 141) of the participants were not on treatment, and among individuals receiving treatment, over 80% (n = 128) sought healthcare from non-public healthcare provider. Over 90% (n = 144) of those who sought care for blood pressure treatment incurred some OOPs. Expected cost for seeking complete care for hypertension per month was over ₹ 306 (> $4.5) and relative expenditure was 13.5% of the MPCE (**Table 1**). Further analysis revealed that the median of relative expenditure was higher for those seeking care from non-public healthcare facility (median: 10.7%) compared to the public healthcare establishments (2.1%). Purchase of medicines (47.7%) accounted for the largest median share of OOPs in non-public healthcare, while it was for transport and other costs (51.3%) followed by purchase of medicines (37.5%) in the public healthcare facilities (**Fig 3**).

**Table 1:** Characteristics of the study participants (N = 300).

Background characteristics	N	Mean or Percentage (95% CI)
**Age**	300	55.99 (54.58–57.41)
**Total expected cost of seeking complete care for hypertension in a month(**₹**)** *	159	306.49 (257.65–355.33)
**Relative expenditure (%) for treatment of hypertension with respect to MPCE** *	159	13.52 (11.13–15.90)
**Age group (years)**		
< 50	101	33.67 (28.29–39.04)
50–63	107	35.67 (30.22–41.12)
>63	92	30.67 (25.42–35.91)
**Education**		
Completed Secondary or above	48	16.00 (11.83–20.17)
Completed Upper-primary	46	15.33 (11.23–19.43)
Completed Primary	56	18.67 (14.23–23.10)
Illiterate/Below primary	150	50.00 (44.31–55.69)
**Sex**		
Female	183	61.00 (55.45–66.55)
Male	117	39.00 (33.45–44.55)
**Social group**		
Others	140	46.67 (40.99–52.34)
OBC	42	14.00 (10.05–17.95)
SC/ST	118	39.33 (33.77–44.89)
**Religion** ^**+**^		
Hinduism	225	75.25 (70.33–80.17)
Islam	74	24.75 (19.83–29.67)
**Civil status**		
Living with partner	195	65.00 (59.57–70.43)
Not living with partner	105	35.00 (29.57–40.43)
**Occupation**		
Service/Business	65	21.67 (16.98–26.36)
Labourer	47	15.67 (11.53–19.80)
Homemaker/Retired/ Student	160	53.33 (47.66–59.01)
Unemployed	28	9.33 (6.02–12.64)
**Economic Class**		
Richest	75	25.00 (20.07–29.93)
Upper Middle	79	26.33 (21.32–31.35)
Lower-middle	70	23.33 (18.52–28.15)
Poorest	76	25.33 (20.38–30.28)
**Duration of Hypertension (years)**		
<5	141	47.00 (41.32–52.68)
≥5	106	35.33 (29.89–40.77)
Not sure/don’t know	53	17.67 (13.32–22.01)
**Co-morbidity**		
No	240	80.00 (75.45–84.55)
Yes	60	20.00 (15.45–24.55)
**Regular treatment for hypertension**		
On regular consultation & medication	71	23.67 (18.83–28.50)
On regular medication only	39	13.00 (9.17–16.83)
On irregular treatment	49	16.33 (12.13–20.54)
Not on treatment	141	47.00 (41.32–52.68)
**Place of treatment for hypertension** *		
Public	31	19.50 (13.27–25.72)
Non-public	128	80.50 (74.28–86.73)
**Health care provider** *		
Public physician	30	18.87 (12.72–25.02)
Private physician	63	39.62 (31.94–47.31)
AYUSH doctor/ Other	19	11.95 (6.85–17.05)
Informal healthcare provider	47	29.56 (22.39–36.73)
**OPP** *		
Absent	15	9.43 (4.84–14.03)
Present	144	90.57 (85.97–95.16)

₹: Indian National Rupee; CI: Confidence Interval; OBC: Other backward classes; SC: Scheduled caste; ST: Scheduled tribe; AYUSH: *Ayurveda*, Yoga and Naturopathy, *Unani*, *Siddha* and Homoeopathy; OOPs: Out of Pocket Payments; MPCE: Monthly per capita expenditure.

* Sample characteristics is based on 159 participants representing patients seeking treatment for hypertension.

^+^ One person did not share information on religion.

Fifteen individuals were reported incurring no OOPs for the usual treatment for hypertension. Majority (n = 9) were female, aged between 50 to 63 years (n = 9), Hindu (n = 12), general caste (n = 9) with below primary or no formal education (n = 10), home maker/ retired (n = 10) and belongs to upper-middle class (n = 6) of the economic strata of the study population.

**Table 2** shows lower odds of having OOPs among participants aged 50–63 years and 63 years and above compared to participants below 50 years. Males when compared to females, and homemaker/retired /student, labourer and unemployed when compared to those in service/business had relatively lower odds of incurring any OOPs. Compared to the richest economic class the poorest had lower odds of having any OOPs, in unadjusted model (uOR _poorest_ 0.22 (CI: 0.04–1.21)). Having hypertension for five years or more (uOR 5.14 (CI: 1.39–19.01) and aOR 5.68 (CI: 1.24–25.99)) and seeking treatment from non-public establishments (uOR 26.32 (CI: 6.80–101.93) and aOR 34.33 (CI: 4.82–244.68)) were positively associated with OOPs.

**Table 2:** Factors associated with out of pocket payments: Logistic regression analysis (N = 159).

	Unadjusted		Adjusted	
	OR (95% CI)	p	OR (95% CI)	p
**Age group (years)**				
< 50	1.00		1.00	
50–63	0.14 (0.02–1.13)	0.06	0.14 (0.02–1.38)	0.09
>63	0.29 (0.03–2.60)	0.27	0.47 (0.04–5.88)	0.56
**Education**				
Completed Secondary or above	1.00		1.00	
Completed Upper-primary	0.89 (0.05–15.00)	0.93	0.39 (0.01–12.94)	0.60
Completed Primary	0.33 (0.03–3.41)	0.35	0.20 (0.01–4.22)	0.30
Illiterate/Below primary	0.24 (0.03–2.00)	0.19	0.09 (0.00–2.49)	0.15
**Sex**				
Female	1.00		1.00	
Male	0.70 (0.24–2.10)	0.53	0.08 (0.01–0.71)	0.02
**Social group**				
Others	1.00		1.00	
OBC	2.19 (0.26–18.37)	0.47	3.13 (0.28–34.91)	0.35
SC/ST	1.09 (0.34–3.43)	0.89	3.36 (0.60–18.97)	0.17
**Religion**				
Hinduism	1.00		1.00	
Islam	1.40 (0.37–5.22)	0.62	2.30 (0.40–13.39)	0.35
**Civil status**				
Living with partner	1.00		1.00	
Not living with partner	0.82 (0.28–2.37)	0.71	0.95 (0.25–3.66)	0.94
**Occupation**				
Service/Business	1.00		1.00	
Labourer	0.16 (0.01–1.91)	0.15	0.08 (0.00–1.65)	0.10
Homemaker/Retired/ Student	0.27 (0.03–2.18)	0.22	0.04 (0.00–0.79)	0.03
Unemployed	0.25 (0.02–2.97)	0.27	0.17 (0.01–3.65)	0.26
**Economic Class**				
Richest	1.00		1.00	
Upper Middle	0.27 (0.05–1.42)	0.12	0.47 (0.07–3.04)	0.42
Lower-middle	0.57 (0.08–4.28)	0.59	1.14 (0.12–11.18)	0.91
Poorest	0.22 (0.04–1.21)	0.08	0.40 (0.06–2.91)	0.37
**Co-morbidity**				
No	1.00		1.00	
Yes	0.74 (0.24–2.31)	0.61	0.54 (0.13–2.24)	0.39
**Duration of Hypertension (years)**				
<5	1.00		1.00	
≥5	5.14 (1.39–19.01)	0.01	5.68 (1.24–25.99)	0.03
**Place of treatment seeking for hypertension**				
Public	1.00		1.00	
Non-public	26.32 (6.80–101.93)	<0.01	34.33(4.82–244.68)	<0.01

CI: Confidence Interval; OBC: Other backward classes; SC: Scheduled caste; ST: Scheduled tribe; OR: Odds ratio.

**Fig 4:**
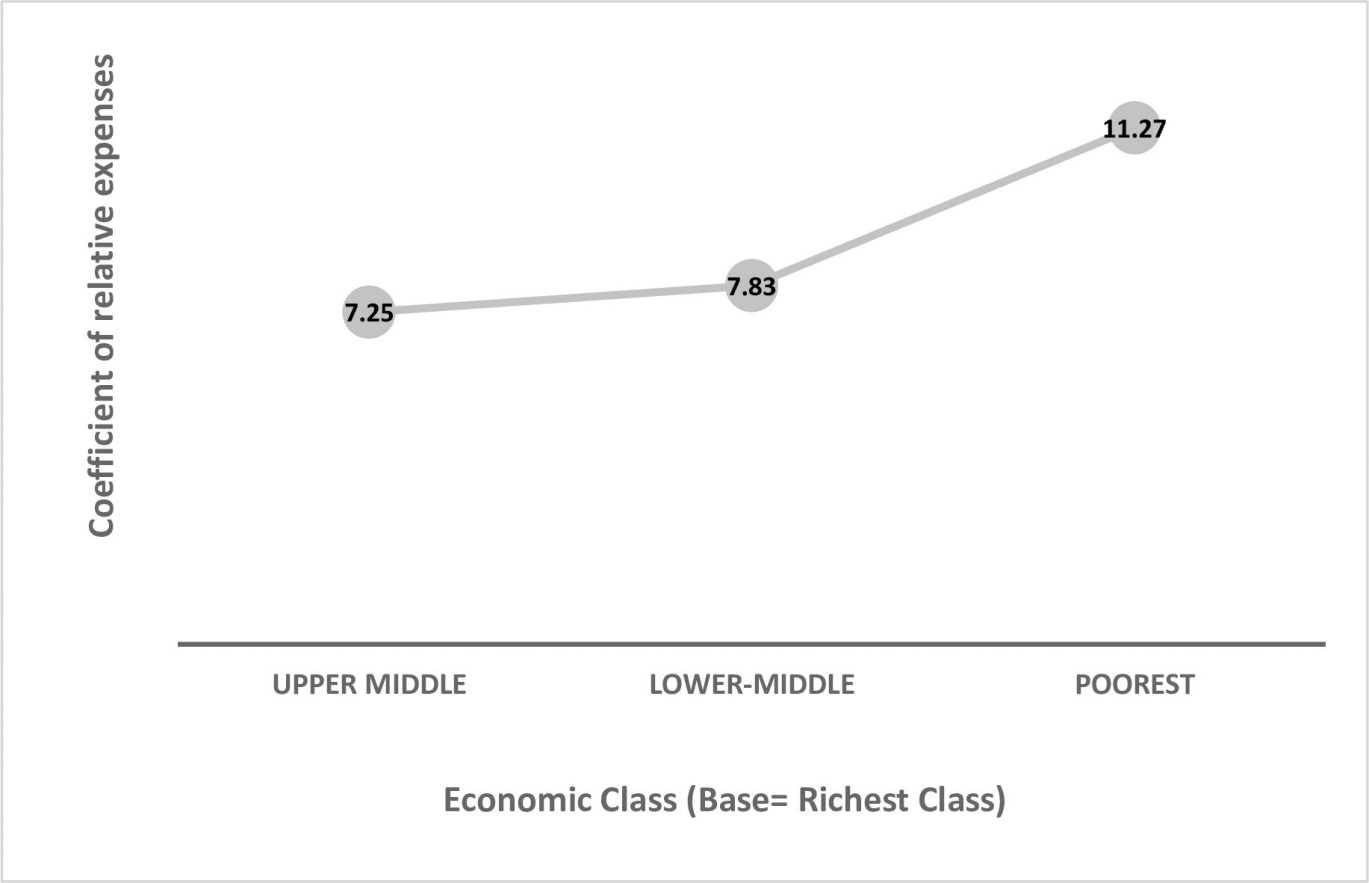
Relative expenditure for care seeking across the economic strata with reference to richest economic class: Findings from linear regression (N = 159).

Linear regression with GLM (**Table 3**) demonstrated lower relative expenditure among people with primary or below level of schooling, compared to highest educational group; (Adjusted Coefficient (aCoeff) _completed primary_ -10.65 (CI: -19.78, -1.51) and aCoeff_no formal education/below primary_ -11.60 (CI: -20.88, -2.32)). The unemployed individuals had more relative expenditure compared to those engaged in service/business (Unadjusted Coefficient (uCoeff)_unemployed_ 8.71 (CI: 0.04,17.38) and aCoeff_unemployed_ 9.34 (CI: -1.74,20.43)). The poorest, lower-middle and upper-middle class had 11, 8 and 7 units of more relative expenditure respectively, compared to the richest economic class (aCoeff_poorest_ 11.27 (CI: 3.82,18.71); aCoeff_lower-middle_ 7.83 (CI: 0.65,15.00) and aCoeff_upper-middle_ 7.25 (CI: 0.80,13.70)) (**Fig 4**). Presence of co-morbidity and visiting non-public establishments both were associated with higher relative expenditure (aCoeff_one or more co-morbidity_ 10.28 (CI: 4.96,15.61); reference group: no co-morbidity and aCoeff_non-public establishment_ 11.55 (CI: 5.74,17.37); reference group: public establishment). Similarly, seeking treatment from private doctors, informal practitioners and AYUSH doctors/others were associated with more relative expenditure (aCoeff_private Doctors_ 18.43 (CI: 12.13, 24.73), aCoeff_informal healthcare provider_ 5.96 (CI: -0.36, 12.28), aCoeff_AYUSH/ Other_ 10.28 (CI: 2.56, 17.99)) when compared to those seeking treatment from government doctors.

**Table 3:** Factors associated with relative expenditure: Linear regression with GLM.

	Unadjusted		Adjusted	
	β (95% CI)	p	β (95% CI)	p
**Age group (years)**				
< 50	0.00		0.00	
50–63	-2.39 (-8.55,3.77)	0.45	-3.22 (-9.84,3.40)	0.34
>63	0.28 (-5.78,6.33)	0.93	-1.66 (-8.51,5.19)	0.64
**Education**				
Completed Secondary or above	0.00		0.00	
Completed Upper-primary	-2.58 (-10.82,5.67)	0.54	-5.17 (-14.14,3.80)	0.26
Completed Primary	-3.15 (-11.02,4.73)	0.43	-10.65 (-19.78,-1.51)	0.02
Illiterate/Below primary	-1.06 (-7.69,5.56)	0.75	-11.60 (-20.88,-2.32)	0.01
**Sex**				
Female	0.00		0.00	
Male	-0.94 (-5.99,4.10)	0.71	-3.39 (-10.90,4.13)	0.38
**Social group**				
Others	0.00		0.00	
OBC	0.43 (-6.95,7.82)	0.91	-2.02 (-9.58,5.55)	0.60
SC/ST	3.44 (-1.78,8.66)	0.20	5.36 (-1.27,11.98)	0.11
**Religion**				
Hinduism	0.00		0.00	
Islam	-0.07 (-5.54,5.40)	0.98	3.23 (-3.37,9.82)	0.34
**Civil status**				
Living with partner	0.00		0.00	
Not living with partner	1.11(-3.68,5.90)	0.65	1.85 (-3.62,7.32)	0.51
**Occupation**				
Service/Business	0.00		0.00	
Labourer	5.57 (-4.40,15.54)	0.27	5.88 (-5.04,16.79)	0.29
Homemaker/Retired/Student	1.59 (-4.38,7.56)	0.60	0.85 (-7.88,9.59)	0.85
Unemployed	8.71 (0.04,17.38)	0.05	9.34 (-1.74,20.43)	0.09
**Economic Class**				
Richest	0.00		0.00	
Upper Middle	5.54 (-0.38,11.46)	0.07	7.25 (0.80,13.70)	0.03
Lower-middle	5.73 (-0.97,12.43)	0.09	7.83 (0.65,15.00)	0.03
Poorest	10.39 (3.82,16.95)	0.00	11.27 (3.82,18.71)	0.00
**Co-morbidity**				
No	0.00		0.00	
Yes	7.74 (2.59,12.89)	0.00	10.28 (4.96,15.61)	<0.01
**Duration of Hypertension (years)**				
<5	0.00		0.00	
≥5	1.64 (-3.10,6.37)	0.50	2.17 (-2.62,6.97)	0.37
**Place of treatment seeking for hypertension**				
Public	0.00		0.00	
Non-public	9.35(3.56,15.14)	0.00	11.55 (5.74,17.37)	<0.01
**Health care provider**				
Public physician	0.00		0.00	
Private physician	14.38 (8.24,20.51)	<0.01	18.43 (12.13,24.73)	<0.01
AYUSH doctor/Other	5.39 (-2.72,13.50)	0.19	10.28 (2.56,17.99)	0.01
Informal healthcare provider	3.40 (-3.07,9.86)	0.30	5.96 (-0.36,12.28)	0.06

GLM: Generalised Linear Model; CI: Confidence Interval; OBC: Other backward classes; SC: Scheduled caste; ST: Scheduled tribe; AYUSH: *Ayurveda*, Yoga and Naturopathy, *Unani*, *Siddha* and Homoeopathy; OPP: Out of Pocket Payments; β: Coefficient.

## Discussion

India has witnessed an increasing burden of hypertension, which demands urgent attention from the public health researchers, program and policy makers. To add on to the existing body of literature on prevention of hypertension in India, this study aims to understand characteristics of healthcare seeking and financial protection among hypertensive population in West Bengal, India. The state of West Bengal recorded nearly 25% of total annual deaths and 13% of disability adjusted life years (DALYs) attributed to hypertension [15, 31]. This study revealed poor health care seeking behaviour, preference of non-public health facilities and high OOPs among patients who sought care for hypertension. Regression analysis adjusted for potential covariates indicate that OOPs are associated with age, sex, occupation, duration of hypertension, and place of treatment seeking for hypertension, while relative expenditure is associated with education, occupation, economic class, comorbidity, place of treatment and healthcare provider.

The population under study were relatively older, female predominated, had low education level, and majority were retired/homemaker. This distribution was similar to other studies where hypertension prevalence was more among elderly, females, and in poor socio-economic strata [32, 33]. The findings of poor health care seeking for blood pressure control, was perhaps due to lack of awareness, affordability and availability of health care services as evidenced from the findings of COHESION study but not elaborated here. Among the hypertensives seeking treatment, OOPs were extensively reported. This scenario corroborates with previous findings of sub-optimal health system response for blood pressure control care [10, 11, 13, 14, 20, 34–37]. However better system response was associated with substantial improvement in indicators like awareness, treatment and control of hypertension in a few developed countries [38]. Similar to other studies, non-public establishments were major places for seeking treatment and public institutions played a minor role for management of hypertension. Similarly, majority sought consultation from private physicians and informal healthcare providers [13, 20, 22]. The presence of OOPs and extent of relative expenditure varied between service utilization from public to non-public health facilities as well as with different service providers. The findings related to OOPs in this study are in line with previous reports including a report of the WHO, Study on global AGEing and adult health (SAGE) but the significant variation observed in OOPs across public and non-public institutions in this study is found to be novel [19, 20]. Earlier studies found medicine purchase as the major share for OOPs [19–21] which corroborates with the findings from this study, however transport and other costs are also found to impose a substantial share of OOPs in public set-up, possibly indicating better accessibility for the non-public establishments in local level compared to public institutions. This could also justify the increased usage of non-public facilities for hypertension management. Contrasting with findings from other studies, the present study reported lower incident of OOPs among male and those belonging to 50 years or above age group [10, 20]. More relative expenditure was associated with higher level of education, whereas it was found to be inversely related with disadvantageous economic class. These findings point towards potential issues of social justice and inequity which share a complex interrelationship [19, 20]. This might be related to poor treatment seeking behaviour among patients with low education and economic status (jointly the lower socio-economic class) owing to low awareness, financial constraint and limited access to healthcare, which may have led to lower possibility of having OOPs. But despite these barriers, patients who sought treatment experienced inequitable financial burden. Similar explanation may be applied for the unemployed group, having more extent of relative expenditure while seeking care but lower odds of OOPs. Lower OOPs among homemaker/retired individuals was perhaps due to better utilization of public health facilities, compared to the service holders/businessmen who generally have less opportunity to visit public outpatient services due to its fixed schedule. Longer duration of hypertension and existence of comorbid conditions require more intense therapy resulting in more possibility of having OOPs and more relative expenditure [10].

The existing national health program NPCDSC, for prevention and control of NCDs, offers provision of treatment for hypertensives along with other NCDs. In the state of West Bengal, the NCD clinics operational under all public health facilities offers consultation and commonly prescribed medication at free of cost. Despite that, we observed poor preference to public establishment and people visiting non-public establishments with more OOPs. The present study indicated that the major share of OOPs in the public facilities were due to transport and other costs (51.28%). Decentralization of NCD clinics up to Health Sub-centre level, mobile clinics may invite more individual to seek care from public facilities including those who are not seeking treatment for accessibility. Study also suggests a substantial amount of OOPs from medicine purchase even in public facilities. Local public health system must ensure a continuous logistic supply including medicines to mitigate the issue of affordability among care seeker. Responsiveness of the respective health system might also play some role which justify preference towards non-public facilities. Findings from this study indicates further research on health care seeking pattern with objectives to understand the reason of specific pattern.

Limitation of the study should be interpreted in light of the results. Firstly, being a cross-sectional study, temporal ambiguity cannot be ruled out. Secondly, as most of variables under study are information based on recall, some chances for recall errors may be present. Thirdly, measurement of exact expenditure and assessing economic status could be debated. To counter the variability of health care seeking, health care expenditure related to hypertension management was calculated as expected cost for having complete care. This may have over-represented the relative expenditure for treatment to some extent. Effects of residual confounding also cannot be ruled out. Within purview of limitations, considering the geographic and demographic uniqueness of the Birbhum population, the findings of this study should be interpreted cautiously for other settings. Despite these limitations, the study contributes tremendously to the existing literature in terms of unique study setting and use of pre-tested and validated study tools. The findings from the study suggest suboptimal financial protection of population for hypertension care. The aspect of awareness generation and evaluation of existing programs on NCDs might be needed for a better financial protection mechanism to people with hypertension.

## Abbreviations

aOR: adjusted odds ratio
BIRPOP: Birbhum Population Project
CI: confidence interval
NCDs: non-communicable diseases
OOPs: out of pocket payments
OR: odds ratio
uOR: unadjusted odds ratio

## References

[1] Abubakar I, Tillmann T, Banerjee A. Global, regional, and national age-sex specific all-cause and cause-specific mortality for 240 causes of death, 1990–2013: a systematic analysis for the Global Burden of Disease Study 2013. Lancet. 2015;385(9963):117–71. doi: 10.1016/S0140-6736(14)61682-2 25530442 PMC4340604

[2] Habib SH, Saha S. Burden of non-communicable disease: global overview. Diabetes & Metabolic Syndrome: Clinical Research & Reviews. 2010;4(1):41–7.

[3] Murray CJ, Ezzati M, Flaxman AD, Lim S, Lozano R, Michaud C, et al. GBD 2010: design, definitions, and metrics. Lancet. 2012;380(9859):2063–6. doi: 10.1016/S0140-6736(12)61899-6 23245602

[4] World Health Organization. The top 10 causes of death 2020 [updated 9 December 2020; cited 2021, 13 September]. [Available from: https://www.who.int/news-room/fact-sheets/detail/the-top-10-causes-of-death].

[5] World Health Organization. Hypertension 2021 [updated 25 August 2021; cited 2021, 13 September]. [Available from: https://www.who.int/news-room/fact-sheets/detail/hypertension].

[6] Alshamsan R, Lee JT, Rana S, Areabi H, Millett C. Comparative health system performance in six middle-income countries: cross-sectional analysis using World Health Organization study of global ageing and health. Journal of the Royal Society of Medicine. 2017;110(9):365–75. doi: 10.1177/0141076817724599 28895493 PMC5987910

[7] Feng XL, Pang M, Beard J. Health system strengthening and hypertension awareness, treatment and control: data from the China Health and Retirement Longitudinal Study. Bull World Health Organ. 2014;92(1):29–41. doi: 10.2471/BLT.13.124495 24391298 PMC3865551

[8] Ibrahim MM, Damasceno A. Hypertension in developing countries. Lancet. 2012;380(9841):611–9. doi: 10.1016/S0140-6736(12)60861-7 22883510

[9] Peck R, Mghamba J, Vanobberghen F, Kavishe B, Rugarabamu V, Smeeth L, et al. Preparedness of Tanzanian health facilities for outpatient primary care of hypertension and diabetes: a cross-sectional survey. Lancet Glob Health. 2014;2(5):e285–92. doi: 10.1016/S2214-109X(14)70033-6 24818084 PMC4013553

[10] Wang Q, Fu AZ, Brenner S, Kalmus O, Banda HT, De Allegri M. Out-of-pocket expenditure on chronic non-communicable diseases in sub-Saharan Africa: the case of rural Malawi. PLoS One. 2015;10(1):e0116897. doi: 10.1371/journal.pone.0116897 25584960 PMC4293143

[11] World Health Organization. Impact of out-of-pocket payments for treatment of non-communicable diseases in developing countries: a review of literature. 2011.

[12] Bovet P, Gervasoni JP, Mkamba M, Balampama M, Lengeler C, Paccaud F. Low utilization of health care services following screening for hypertension in Dar es Salaam (Tanzania): a prospective population-based study. BMC Public Health. 2008;8(1):407. 19087300 10.1186/1471-2458-8-407 PMC2615777

[13] Baliga SS, Gopakumaran PS, Katti SM, Mallapur MD. Treatment seeking behavior and health care expenditure incurred for hypertension among elderly in urban slums of Belgaum City. Community Med. 2013;4(2):227–30.

[14] Anchala R, Kannuri NK, Pant H, Khan H, Franco OH, Di Angelantonio E, et al. Hypertension in India: a systematic review and meta-analysis of prevalence, awareness, and control of hypertension. J Hypertens. 2014;32(6):1170–7. doi: 10.1097/HJH.0000000000000146 24621804 PMC4011565

[15] Indian Council of Medical Research; Public Health Foundation of India and Institute for Health Metrics and Evaluation. India: Health of Nation’s States—The India State- level Disease Burden Initiative. New Delhi, India: ICMR, PHFI and IHME; 2017. 2017.

[16] Gupta R, Gaur K, CV SR. Emerging trends in hypertension epidemiology in India. J Hum Hypertens. 2019;33(8):575–87. doi: 10.1038/s41371-018-0117-3 30254382

[17] Directorate General of Health Services; Ministry of Health & Family Welfare Government of India. National Programme for Prevention and Control of Cancer, Diabetes, Cardiovascular Diseases and Stroke (NPCDCS) 2017 [updated 26 August 2020; cited 2021 February 22]. [Available from: http://dghs.gov.in/content/1363_3_NationalProgrammePreventionControl.aspx].

[18] Ministry of Health & Family Welfare GoI. National Health Policy 2017 [cited 2021 February 22]. [Available from: https://www.nhp.gov.in/nhpfiles/national_health_policy_2017.pdf].

[19] Bhojani U, Thriveni B, Devadasan R, Munegowda C, Devadasan N, Kolsteren P, et al. Out-of-pocket healthcare payments on chronic conditions impoverish urban poor in Bangalore, India. BMC Public Health. 2012;12(1):990. doi: 10.1186/1471-2458-12-990 23158475 PMC3533578

[20] Brinda EM, Kowal P, Attermann J, Enemark U. Health service use, out-of-pocket payments and catastrophic health expenditure among older people in India: The WHO Study on global AGEing and adult health (SAGE). J Epidemiol Community Health. 2015;69(5):489–94. doi: 10.1136/jech-2014-204960 25576563

[21] Engelgau MM, Karan A, Mahal A. The Economic impact of Non-communicable Diseases on households in India. Global Health. 2012;8(1):9. doi: 10.1186/1744-8603-8-9 22533895 PMC3383461

[22] Kanungo S, Mahapatra T, Bhowmik K, Saha J, Mahapatra S, Pal D, et al. Patterns and predictors of undiagnosed and uncontrolled hypertension: observations from a poor-resource setting. J Hum Hypertens. 2017;31(1):56–65. doi: 10.1038/jhh.2016.30 27193382

[23] Ghosh S, Barik A, Majumder S, Gorain A, Mukherjee S, Mazumdar S, et al. Health & Demographic Surveillance System Profile: The Birbhum population project (Birbhum HDSS). Int J Epidemiol. 2015;44(1):98–107. doi: 10.1093/ije/dyu228 25540150

[24] Chobanian AV, Bakris GL, Black HR, Cushman WC, Green LA, Izzo JL Jr., et al. The Seventh Report of the Joint National Committee on Prevention, Detection, Evaluation, and Treatment of High Blood Pressure: the JNC 7 report. JAMA. 2003;289(19):2560–72. doi: 10.1001/jama.289.19.2560 12748199

[25] Ghosh S, Mukhopadhyay S, Barik A. Sex differences in the risk profile of hypertension: a cross-sectional study. BMJ Open. 2016;6(7):e010085. doi: 10.1136/bmjopen-2015-010085 27466234 PMC4964242

[26] Baker RPJSSCR. New technology in survey research: Computer-assisted personal interviewing (CAPI). 1992;10(2):145–57.

[27] Howe LD, Galobardes B, Matijasevich A, Gordon D, Johnston D, Onwujekwe O, et al. Measuring socio-economic position for epidemiological studies in low- and middle-income countries: a methods of measurement in epidemiology paper. Int J Epidemiol. 2012;41(3):871–86. doi: 10.1093/ije/dys037 22438428 PMC3396323

[28] Das J, Chowdhury A, Hussam R, Banerjee AV. The impact of training informal health care providers in India: A randomized controlled trial. Science (New York, NY). 2016;354(6308). doi: 10.1126/science.aai9379 27846471

[29] Glymour MM, Greenland S. Causal Diagrams. In: Rothman KJ, Greenland S, Lash TL, editors. Modern epidemiology. 3 ed. New Delhi: Wolters Kluwer (India) Pvt Ltd; Eighth Indian Reprint 2016. p. 183–209.

[30] Matsaganis M, Mitrakos T, Tsakloglou P. Modelling health expenditure at the household level in Greece. The European Journal of Health Economics. 2009;10(3):329–36. doi: 10.1007/s10198-008-0137-y 19037671

[31] Institute for Health Metrics and Evaluation (IHME). GBD India Compare | Viz Hub 2019 [cited 2021 February 22]. [Available from: https://vizhub.healthdata.org/gbd-compare/india].

[32] Irazola VE, Gutierrez L, Bloomfield GS, Carrillo-Larco RM, Dorairaj P, Gaziano T, et al. Hypertension Prevalence, Awareness, Treatment, and Control in Selected Communities of Nine Low-and Middle Income Countries: Results From the NHLBI/UHG Network of Centers of Excellence for Chronic Diseases. Global heart. 2016;11(1):47. doi: 10.1016/j.gheart.2015.12.008 27102022 PMC4843831

[33] Sarki AM, Nduka CU, Stranges S, Kandala NB, Uthman OA. Prevalence of Hypertension in Low- and Middle-Income Countries: A Systematic Review and Meta-Analysis. Medicine (Baltimore). 2015;94(50):e1959. doi: 10.1097/MD.0000000000001959 26683910 PMC5058882

[34] Chow CK, Teo KK, Rangarajan S, Islam S, Gupta R, Avezum A, et al. Prevalence, awareness, treatment, and control of hypertension in rural and urban communities in high-, middle-, and low-income countries. JAMA. 2013;310(9):959–68. doi: 10.1001/jama.2013.184182 24002282

[35] Kaur P, Rao SR, Radhakrishnan E, Rajasekar D, Gupte MD. Prevalence, awareness, treatment, control and risk factors for hypertension in a rural population in South India. Int J Public Health. 2012;57(1):87–94. doi: 10.1007/s00038-011-0303-3 21947549

[36] Singh AK, Kalaivani M, Krishnan A, Aggarwal P, Gupta SK. Prevalence, awareness, treatment and control of hypertension among elderly persons in an urban slum of Delhi, India. Indian Journal of Medical Specialities. 2014;5(1):7–10.

[37] Tocci G, Ferrucci A, Pontremoli R, Ferri C, Rosei E, Morganti A, et al. Blood pressure levels and control in Italy: comprehensive analysis of clinical data from 2000–2005 and 2005–2011 hypertension surveys. Journal of human hypertension. 2015;29(11):696. doi: 10.1038/jhh.2015.4 25673112

[38] Joffres M, Falaschetti E, Gillespie C, Robitaille C, Loustalot F, Poulter N, et al. Hypertension prevalence, awareness, treatment and control in national surveys from England, the USA and Canada, and correlation with stroke and ischaemic heart disease mortality: a cross-sectional study. BMJ Open. 2013;3(8):e003423. doi: 10.1136/bmjopen-2013-003423 23996822 PMC3758966

